# Gerontechnology: Providing a Helping Hand When Caring for Cognitively Impaired Older Adults—Intermediate Results from a Controlled Study on the Satisfaction and Acceptance of Informal Caregivers

**DOI:** 10.1155/2012/401705

**Published:** 2012-03-22

**Authors:** Anelia Mitseva, Carrie Beth Peterson, Christina Karamberi, Lamprini Ch. Oikonomou, Athanasios V. Ballis, Charalampos Giannakakos, George E. Dafoulas

**Affiliations:** ^1^North Denmark EU-Office, Aalborg Municipality, Boulevarden 13, 9000 Aalborg, Denmark; ^2^Center for TeleInFrastruktur (CTiF), Aalborg University, Niels Jernes Vej 12, 9220 Aalborg, Denmark; ^3^Municipal Enterprise for Social Development (DEKA) of Trikala, European Projects Department, Valkanou 6, 42100 Trikala, Greece; ^4^e-Trikala SA, e-Health Department and R&D Department, Street Sarafi 44, 42100 Trikala, Greece; ^5^Hellenic Statistical Authority, Census Department, Piraeus 46 & Eponiton, 18510 Piraeus, Greece

## Abstract

The incidence of cognitive impairment in older age is increasing, as is the number of cognitively impaired older adults living in their own homes. Due to lack of social care resources for these adults and their desires to remain in their own homes and live as independently as possible, research shows that the current standard care provisions are inadequate. Promising opportunities exist in using home assistive technology services to foster healthy aging and to realize the unmet needs of these groups of citizens in a user-centered manner. ISISEMD project has designed, implemented, verified, and assessed an assistive technology platform of personalized home care (telecare) for the elderly with cognitive impairments and their caregivers by offering intelligent home support services. Regions from four European countries have carried out long-term pilot-controlled study in real-life conditions. This paper presents the outcomes from intermediate evaluations pertaining to user satisfaction with the system, acceptance of the technology and the services, and quality of life outcomes as a result of utilizing the services.

## 1. Introduction

Dementia is a group of syndromes associated with a loss of memory and other intellectual functions that are serious enough to interfere with daily task performance. There are around 40 types, or causes, of dementia, the most widely known being Alzheimer's disease. Dementia is commonly associated with aging as the risk for exhibiting symptoms of dementia increases with age, nearly doubling every 5 years after the age of 60. The chances of having dementia over the age of 65 are one in 50 and that increases to one in 5 for those over 80 years and up to 50% in adults over age 85 [1, 2]. Mild cognitive impairment (MCI or CI) is closely related to dementias. Plassman et al. [3] found in their follow-up study that of their participants who had CI and no dementia, nearly 12% advanced to dementia annually. Although MCI is often viewed as a precursor to developing dementia, the relationship is not fully understood.

With advancements in medicine, economy, and technology, the human lifespan has increased significantly over the last decades; additionally, dementia rates have increased [4] and this poses questions about the *quality* of extended years of life. In the context of ISISEMD project, we use quality of life (QOL) to mean “the individual's perception and evaluation of the impact that the disease and its consequences have produced in their lives” [5]. As there is no cure for dementia and current treatments serve only to reduce the debilitating effects, a major area of focus in this type of caregiving is on maintaining or increasing patient QOL and reducing care-related stress.

By and large, the majority of informal caregiving is carried out by persons (usually family members, close friends, or neighbors) who have not had any formal training in caregiving and do not receive any economic retribution for their tasks. Both as individuals and as society as a whole, it is not possible to pay for the costs that informal caregiving mitigates from formal caregiving services. Needless to say, informal caregivers provide an invaluable service that is impossible to remunerate; they are the backbone of long-term care. To be a family caregiver for an older adult with cognitive impairment is a heavy task that comes with financial implications for the individual families as well as society, as well as the majority of the cases resulting in increased emotional and physical stress. 

Aging in one's own home is a growing demand among baby boomers and the related technological solutions are market estimated at €14 Billion by 2020 [6]. The idea of *aging in place* is not a new one, yet only recently have governments and organizations started working towards incorporating appropriate solutions, one of the main modes is through information and communication technology (ICT) for aging, also known as gerontechnology. The inspiration of tele home care is to have the home environment modified in order to meet the increasing care and safety needs and to reduce forced relocation, which often leads to transfer trauma and relocation stress syndrome. Instead of older adults selling their homes and moving into retirement communities or care institutions, home modifications allow the living environment to become user-friendly to aging adults [7]. The European Commission (EC), in collaboration with the Member States, has recently put more focus on the coming challenges of care for older adults with this type of disability living in their homes. Likewise, many governments are now focusing on ICT systems for supporting the elderly and chronically ill to live as independently as possible and to help citizens to be treated and/or cared for safely in their own homes. The European Commission has supported a Policy Support Program (PSP) pilot study exploring the use of Assistive Technologies (ATs) for the care of older adults with cognitive impairment (CI) by co-funding the ISISEMD project. ISISEMD has taken a holistic approach—examining and meeting the needs of the elder/caregiver dyad as a whole. Project ISISEMD (intelligent system for independent living and self-care of seniors with cognitive problems or mild dementia) [8] concentrates on adults over the age of 60 who have a documented history of CI and their caregivers. The project has developed and tested an innovative set of scalable technologies for the purpose of easing the caregiving and care receiving activities associated with dementia care and to have a positive effect on users' QOL. The project was 30-months in duration from March, 2009 to August, 2011, and involved 12 partners representing end-user organizations (Municipality of Frederikshavn—Elderly Care Department from Denmark, Belfast Health, and Social Care Trust from UK, Municipality of Trikala from Greece, Municipality of Lappeenranta-Health and Social Care Department from Finland), industrial organizations (Hewlett Packard-Italy, Alcatel-Lucent-Italy), SMEs (Converge ICT Solutions from Greece, Eltronic from Denmark, Socrate Medical from Italy ), academia (Aalborg University from Denmark, National Technology University of Athens from Greece), and one public office (North of Denmark EU-Office from Denmark). During the first year of the project, the focus was to design, adjust, and implement the services which were installed in participants' homes during the second year. The services were tested in four European countries (Denmark, Finland, Greece, and UK) in real-life conditions and with the three main end-user groups–elderly with CI, their informal caregivers, and the formal caregivers. 

ATs for dementia care in the ISISEMD project encompass a broad range of devices that currently exist on the market, from a touch screen computer and sensors to determine ambient functions such as temperature, movement, fire/smoke, cooking activity to Global Positioning Systems (GPS) to determine where a person is in real time. The aim is to use accessible technologies in a new way so that the user-friendliness and interoperability of such systems is increased. The goal of all, however, is to maintain or increase the safety of the person living with CI and to have a positive effect on the QOL of all involved. 

The contribution of this paper is to present results from a controlled study carried out in real-life conditions with older adults with CI and their informal caregivers (ICGs) from four European pilot sites. Their homes were installed with the technical equipment that gives them and their informal caregivers possibility to use innovative home support services for a period of more than six months. The controlled study was carried out for 15 months, with intermediate and final evaluations. Results from the intermediate evaluations of the services are presented in this paper, with a focus on informal caregivers and from user acceptance and satisfaction of the technologies view point. Moreover, an analysis of the influence on the QOL and the stress of caregiving for the family caregivers are provided. 

The rest of the paper is organized as follows: in Sections 2 and 3, we give an overview of the experience and stress of caregiving and how the ISISEMD services can help the informal caregiver and their relative with CI. In Section 4 we shortly present different aspects of the assessment methods. The main contributions in this paper are presented in Section 5—primary and secondary outcomes from intermediate evaluations for QOL, stress of caregiving and user satisfaction and acceptance of the AT services. The paper ends with discussions of the findings in Section 6 and conclusions in Section 7. As a qualitative feedback from the controlled study, the voice, and perception of test persons and their informal caregivers is presented in the appendix by “success stories” from using the home services. 

## 2. Caregivers and Society Experience Caregiving Stress in Dementia

Individuals who have conditions resulting in chronic illness or disability often face challenges in carrying out their daily activities, such as meal preparation, bathing or transportation, which involves a considerable amount of time, devotion, perseverance, and patience to perform these tasks. In most dementia situations around the world, caregiving is carried out informally by family and friends. Statistically, the majority of ICGs are female family members and most often the middle-aged child or spouse of the person with CI [9, 10]. Assistance to the person with dementia is generally provided by a single caregiver and this responsibility lasts for an average of 5 years [11]. Caregiving is typically delivered (as long as possible) in the residence of the dependent person on a continual basis, ranging from personal safety and psychological support to physical care, such as hygiene. Members of the caregiving/supporting team determine who will provide care and in which roles and what resources are available; however, caregiving for dementia is rarely a straightforward and static arrangement. The total hours of caregiving per week can easily exceed most national standards for full-time employment, especially in the advanced stages of the process, as the person becomes increasingly dependent and requires daily, continuous assistance. For many caregivers, the requirement of full-time help often results in a decline in the ICGs personal and professional life, ability to manage the household(s), and to perform the range of care and personal activities. 

It is well documented that the caregivers of persons with dementia experience substantial stress from the caregiving tasks they perform [9] and the need for permanent care to the chronically disabled person often leads to a decrease in the QOL of caregivers [12, 13]. De Vugt et al. [14], Bauer et al. [15], and others in the field report negative effects on ICGs QOL when compared to noncaregivers. According to Deeken [16], the stressors and their resulting effects on ICGs also influence when and why ICGs seek out formal caregiving (including institutionalization). In 2008, Adams [17] reported that caregivers are more likely to experience depression, anxiety, and physical health issues when compared to non-caregivers, while also reporting less hope for the future, less happiness, less enjoyment of life and greater degrees of sadness, being bothered, and loneliness. Since CI and dementia are typically chronic in nature and there is no determined end point of the care needs, caregiving is correlated to reduced physical, psychological and social health [18]. Due to research in this area, informal caregiving is respected as a critical stress factor; this is one area of focus that ISISEMD is developing solutions for. It is not uncommon that ICGs often experience greater measurable benefits from the use of AT than those with dementia themselves. A commonly accepted theory for this phenomenon is that awareness and insight into the situation is one major factor influencing fluctuations in QOL, and where those with decreased cognition experience an increasing lack of awareness and insight, it becomes more difficult for them to assess their QOL status. Meanwhile, the ICGs (typically) maintain their awareness of the situation and thus experiences greater stress from its increasing demands as the illness progresses. Furthermore, due to the increasing stress for ICGs, they often project negative attitudes on to the elderly persons (EP) QOL and typically rate their loved one's QOL as lower than the person would rate it themselves. This may be due to evaluating EPs QOL as ICG sees it versus as how EP sees it or to anticipating that with increasing need for care, QOL will decline. The exact nature of these observations is confounded and an area of focus in gerontology and caregiving research that ISISEMD hopes to provide insight to. 

It is well known that a disabled person may often disorganize the life of the family, disrupting the previous balance; it is also well known that caregivers of persons with dementia experience substantial stress from their caregiving tasks. The concept of the “burden of care” was defined by the American gerontologist Zarit in 1985 [19], as the discomfort encountered by the primary caregiver of an older family member during their caregiving duration. Caregiver burden includes the caregiver's health, psychological wellbeing, finances and social life, among others. Since 1985, there have been numerous studies demonstrating the negative impact of increased caregiver stress on the person with dementia as well as caregiver's overall health [9]. During ISISEMD project, we have observed that the term “burden” has a negative connotation and impact on the family caregivers who care for their relative because they care deeply for them and choose to carry out these activities themselves. However, as caregiving inherently causes extra stress in their everyday life and this is one of the parameters measured during the project, we use the term “stress” and not “burden” as a more accurate and respectful terminology.

By introducing ATs to the care situation, ISISEMD project aims to have a positive impact on the informal caregivers and the society as a whole; below, we list the foreseen added value for them. 

Foreseen value for the ICGs as a result of using ISISEMD technology services:

- reduction or maintenance of the level of the caregiving stress;
- information on the location of their EP when outdoors;
- interaction with the services is easy to manage and with high level of individualization, accommodating ICGs who may not have previous experience with computers, also offering initial default settings;
- means to communicate with EP remotely (through video service, mobile phone, or Lommy);
- increased feeling of safety regarding the living environment of EP;
- increased feeling of satisfaction in caregiving responsibilities;
- ability to allocate more time for example personal hobbies and social activities.

Foreseen value for society as a result of using ISISEMD technology services:

- financial diminution by saving time and expenses for traveling to and from EP home and for making telephone calls;
- reduced health and social care costs for the local communities;
- transfer of tasks between caregivers, which includes untrained ICGs to be able to perform caregiving responsibilities with enhanced or new methods;
- ability for the formal caregivers (FCGs) to provide better quality care and/or care for more clients as tasks become less demanding through the technology;
- creation of business opportunities;
- influence on health and social care policies.

Most gerontologists understand caregiving stress to be an outcome of the caregiving situation and as being dependent upon variables presented in the situation and in the caregiving relationship. These stressors are also influenced by the coping strategies of the caregivers. As mentioned, caregiving for a cognitively impaired person, especially a family member, can be straining. It is not uncommon for such caregivers to experience physical symptoms, such as sleep deprivation, lapses in memory themselves (which can also be a sign of depression), or social isolation due to reduced hours spent on free-time activities. There are resources for caregivers, such as respite programs, but these are not accessible or acceptable in every area. They may come with an unwanted stigma or be out of the caregiving budget. These factors may also increase the stress a caregiver experiences in their role. Furthermore, when a threshold for stress is reached, negative behaviors and feelings—however unintentional they may be—can spill over into the caregiving relationship. At the family (micro)level, solutions to support caregiving tasks and health and psychosocial consequences are needed. Research on corroborating programs and services aimed at delivering knowledge, skills and support to the caregiving experience can in turn promote policies (macro level) to ease the strain of caregiving on the individuals as well as society. 

## 3. ISISEMD Services—A Helping (Automated) Hand from Technology 

The ISISEMD services are related to supervising the conditions of the home and any alarming behavior of the person with dementia. The system is nonintrusive, it yet provides a way to alert the elderly themselves as well as caregivers and emergency services if there would be an event that may pose a danger to them. The ISISEMD system is comprised of several technologies that communicate with each other and interoperate on the platform. One of the technical design goals was to utilize existing technologies (computer, GPS device, pressure, and smoke/fire sensors, etc.) and provide a way for them to interact intelligently. This increases the feasibility of introducing new technologies and services to the platform as they will be developed in the future as well as ensures that presently accessible (and affordable) equipment will not necessarily become obsolete as soon as newer technology is available, helping to reduce the costs of purchasing and maintaining the ISISEMD system. As the devices are integrated into one service platform, they operate in an automatic and intelligent manner, recognizing patterns of behavior (e.g., sleep habits and average room temperature) and noting fluctuations that may signal a change in status or potential danger. For the caregivers, this means that they can spend less time and energy worrying and “checking in” on their older adult because they will be alerted by the system when their physical interaction is required. 

There are two categories of equipment in the ISISEMD system: *interactive devices and non-interactive devices.* The majority of the devices in the ISISEMD platform is noninteractive and includes temperature and flood sensors, smoke alarms, electricity monitors for cooking activity, pressure sensors to determine sleeping patterns, and front and fridge door sensors. The interactive devices are chosen or designed to be easy for the end-user to operate; due to the nature of the involvement being a gerontechnological system, masking the intervention was not possible and the telecare platform was visible and interactive to end users. In particular, the devices that the older adult with cognitive impairment operates require minimal interaction or no interaction at all. These include the Carebox (HP Touch Screen computer), Lommy (simple GPS device) with only one button which sends and receives phone calls, and internet connection, which is provided to the end users through the project funds. A number of the functions and services of the platform are easier to access than current mainstream technologies for people with dementia. 

The *interactive devices* are those which exchange contents when prompted by the user and are under the user's control. Examples of interactive devices are for ICG-mobile phone, web portal access on a computer; for EP GPS device and a touch screen. 

The *non-interactive devices* are those that, once installed by a technician, do not require any user manipulation. Examples of non-interactive devices are home automation controllers including flood detectors, and fire sensors. 

**Figure 1:**
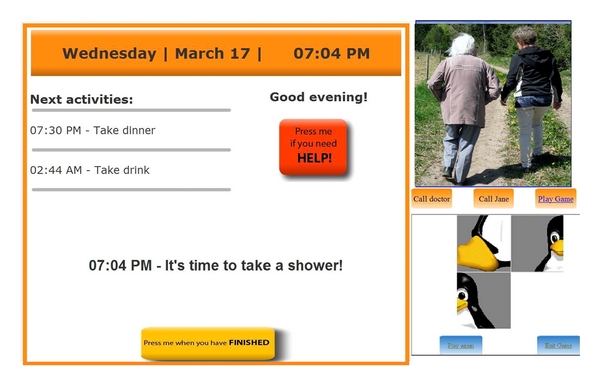
Screen shot of the Carebox with the Help and Confirmation button from the Reminders Service and Memory Lane and Brain Game.

The Carebox Touch Screen serves as the graphical user interface to provide information to EP at home. To accommodate different user needs, there are three levels of interaction of EP with the Carebox: no interaction at all (EP does not need to press any button, just needs to notice the screen from time to time); some interaction via “soft button”—in this case a distinct Help Button is shown on the Carebox that EP can activate; more advanced interaction—additional “soft button” for confirmation of an activity can appear or a Brain game can be played. There is also the possibility to add the Memory Lane service (picture slide show) and a video-call service. The view of the Carebox possesses a high level of flexibility and individualization, too–depending on the health status of EP, the “Help Button,” the Brain games, the Memory Lane, and the video-call service may be removed from it.  Figure 1 depicts the Carebox view with the highest level of interaction. 

The computer of the Carebox also collects data from the sensors installed in the home and the data is sent over the Internet to the ISISEMD web portal. Following the required ethical, privacy, and security requirements, EP designates the caregivers that are allowed access to the web portal, where they can view only the information that is related to that EP. Depending on the user's preferences, the system can automatically send notifications, alerts, and alarms to ICG and FCG via SMS text messaging or email. Almost all possible aspects of the services are easy to be personalized, based on the individual, current needs of both EP and ICG. It also notifies EP via the Carebox Touch screen if there is a dangerous situation in the home.

Automatic services for EP include:

- home and personal safety observations, such as notification if fire/smoke is detected in the home or the front door is left open or a cooking activity is going on for too long;
- reminders and prompts for basic daily activities such as meals and medications;
- predefined reminders exist but there is also the possibility to enter and show personalized “free text reminders,” such as “*Marie* is coming to visit at 15 : 00”;
- locating service when the person is out of the home so that caregivers can find them if they become disoriented;
- reality orientation by displaying the date and time on the Carebox.

Ad hoc services for EP include:

- cognitive stimulation (Brain Games that can be played on the Carebox);
- reminiscence activities (Memory Lane shows a slide show with family pictures on the Carebox);
- video communication with caregivers at the touch of a “soft” button showing telephone image on the Carebox touch screen;
- automatic contact button when in the home (soft “Help” button on the Carebox touch screen);
- automatic contact button when outside the home (red button on the Lommy device).

Automatic services for IGCs include:

- alerts, notifications, and alarm services delivered to mobile phone or email;
- request for help/contact from EP when EP is in the home;
- request for help/contact from EP when EP is outside home, also providing information for the current EP position;
- alert if EP has fallen when outside home.

Ad hoc services for ICGs include:

- through the ISISEMD web portal, ICG can see an overview of daily activities, notifications and alarms raised by the system;
- through the ISISEMD web portal, ICG can see an overview of activity history (lifestyle pattern);
- video-call service for communication with elderly;
- call to the Lommy device when EP is out of the home;
- locating EP outdoor via the Lommy GPS device through the ISISEMD portal.

The following paragraphs give more details about the ISISEMD services, defining them and their role in the home care environment. In total, ISISEMD offers a possibility of 17 services, which can be selected depending on EP and ICG desires. In this way, the system can provide better support via increased care assistance which is accommodating to the progression of the dementia impairments. More technical information for the services is provided in [20]. 

### 3.1. Service Information for the Informal Caregivers

#### 3.1.1. Scheduler

The scheduler enables ICGs to define the parameters (time period, temperature, dangerous versus harmless behaviors, acceptable distance or time away from the home, etc.) which will activate alarms and alerts in accordance with the identified needs of individual elderly person. 

#### 3.1.2. Home Safety

This application facilitates home automation by using alerts, alarms and sensors at the home to facilitate the home safety. Incorporated into this service are kitchen equipment (stove and oven) control, flood, smoke and fire sensors, front door, and other equipment or situations that may pose physical threat.

The electric cooking guard keeps measurements of the time, temperature, and electric current going to the stove and oven. This gives the consumer a predefined time span to use the stove (i.e., 45 minutes), after which, an alert is shown on the Carebox screen and voice notifies the elderly about the cooking activity, giving them the chance to react. If the cooking activities have not stopped after another predefined time period, the system automatically sends an alarm to the informal caregiver. 

#### 3.1.3. Nocturnal Movement Detectors

The bed occupancy sensor generates an alarm if it detects that a user has been out of bed for more than a normal period of time, subject to their individual habits. For example, the sensor can register an alert if EP starts leaving the bed several times during the night. This could signify that they are using the toilet more often—which could require a change in medication, or if they are experiencing nighttime wandering—which could signify a change in cognition, or if a person has not returned to bed—which could indicate they have fallen down while out of bed.

#### 3.1.4. Alerts

An alert is when the caregiver will be notified of the information but a response is not urgent. This information requires some follow-up to be carried out within a preset period of time. This includes: the refrigerator has been left open, front door has been left open, water was left running, the stove has been left on, EP is out of bed for several hours during the night, and so forth. By setting individual parameters through the scheduler, the system is set up to interpret the information transmitted from the sensors and distinguish between a normal activity pattern and unusual activity, which may indicate a change in EP's condition or needs. This enables caregivers to investigate the reason for the change and provide appropriate assistance and amend their support plan accordingly. 

#### 3.1.5. Alarms

Alarms are sent to caregivers in case of events they wish to know of right away, such as if EP has left the house in the middle of the night. The alarm service can be activated automatically and on demand as the alarm sensors are connected to the central unit (from the Carebox computer in the home to the ISISEMD web portal) and a message informs the user about the nature of the alarm. Alternative alarms and message flows with escalation can be foreseen in special cases (e.g., the relative is traveling abroad or is unavailable on their mobile phone) so that critical issues can be addressed properly. The alarm service itself is not only a simple event, notification or message, it can be an overall history of such messages that provides valuable information to the caregivers.

Alarms that require immediate action include:

- door alarms—the house can be equipped with door alarms which can send an alert when the front door is opened (e.g., if EP is not safe to leave the home alone) or if there is unusual activity with the door (e.g., if EP leaves the home at odd hours, such as middle of the night);
- refrigerator and cooking monitoring alarm—this generates an audio alarm for EP when the refrigerator door has been left open for a specific period (e.g., longer than 7 minutes) and automatically sends an alarm to ICG if the door is not closed within a specific period from when the audio alarm is generated (e.g., refrigerator door has been opened 7 minutes and the 1 minute audio alarm to EP has not resolved the issue). The audio alarm is only generated for a specific period and stops once the alarm has been sent to the caregiver. This also enables the recording of a pattern of opening and closing of the fridge to gain perspective of the daily habits, for example, if EP is eating meals at appropriate times. The cooking monitoring alarm is operating on the same principle—after detecting that the stove is on longer than the predefined time period, the system displays an alarm message on the Carebox screen, thus giving the elderly the opportunity to react and turn off the stove or oven. The caregiver receives an alarm only when the cooking appliances are still not turned off;
- smoke, fire, and flood alarms—these signify imminent danger and audio alarms are generated in EPs home as well as alarms sent to caregivers.

### 3.2. Service Information for the Elderly

#### 3.2.1. Reminders on Carebox

The Carebox Touch screen computer installed in the participant's house displays text with reminders and relays attention sounds or prerecorded audio messages. The service is automatic and the user can see the reminder text and hear the attention sound and/or the voice prompt for the activity on the Carebox they are responsible for and enforce the structure of the daily routine. This aspect of the ISISEMD system is as noninteractive as possible, as people with dementia have difficulty learning to use new devices. The alert tells and shows if there is an upcoming appointment, meal, medication, or task. Reminder alerts could be used to prompt EP to take medication, prepare meals, attend an appointment or remind them of planned visitors to their home. Reminders can be automatically repeated multiple times, at short intervals. The Carebox is also automatically showing the next two events that elderly need to perform under a list of next events, but never more than 2 events at a time to avoid invoking anxiety or confusion. Furthermore, the Carebox always displays the date and time to help orient EP.

Caregivers—or possibly the elderly person, if they are able—could make a change to this schedule or enter new reminders and events (i.e., Happy Birthday), which can be accessed via the portal at any time. There is also an option for ICG to request a confirmation to a specific reminder/activity, such as taking medications. In this case another soft button appears on the Carebox screen and if not pressed, the system automatically sends information to ICG (see Figure 1). 

#### 3.2.2. Memory Lane with Personal Pictures on Carebox

This is a picture slide show that is shown on the upper right corner of the Carebox. The ICGs upload pictures and there is the possibility to write a short title to be displayed with each picture and the speed of the picture show can be adjusted. EP does not need to interact with this service but it is hoped that it will stimulate memories, conversations and reinforce their sense of self.

#### 3.2.3. Brain Games on Carebox

This service provides the EP the possibility to play Brain games on the Carebox, such as word finds, appropriate puzzles, etc. The type of game can be selected by formal or informal caregiver via the web portal. 

#### 3.2.4. Video-Call Service

This service is mainly initiated by a caregiver—when a video call is displayed as incoming on the touch screen, EP can hear a traditional telephone ringing sound and can accept the call simply by pressing one “soft button” on the screen. If desired and able, EP can also use this service to make calls. 

#### 3.2.5. Outdoor Safety with the Lommy GPS Device

The required technologies for this service are a GPS positioning system and GPRS communication. The GPS is useful if the client has a tendency to get confused or disoriented when they are outside the home. When EP leaves the home, they take the Lommy device with them. Through this device, they are able to contact their caregiver and can be located should the need arise. The GPS is activated when the elderly person leaves the home, and the caregiver can see their positioning on a map by logging into the ISISEMD portal. By pressing the red button (the only external feature on the Lommy), the Lommy sends a sms with the current position or directly calls the ICG (depending on the individualized settings); alternatively, ICGs can call back the Lommy as a regular telephone number and EP only needs to press the red button to activate the communication.

Alarms—an alarm is generated when EP presses the red contact button; has left the “safe” geographical area; EP has fallen down; and to signify low battery level. The device is about the size of a deck of cards and is fitted to an item the person would normally take with them when they go out, such as in the jacket pocket or purse. Additionally, we have found that placing a large picture of the device near the main door helps to remind EPs to take the device with them.

## 4. Methods of the Assessment

### 4.1. Primary Research Outcome

The project is classified under the PSP Theme 1 (ICT for user-friendly administration, public services, and inclusion) and states a strategic objective of using ICT for aging well with cognitive problems by combining assistive and independent living technologies. Primary research outcomes consist of measuring the impact on QOL and caregiving stress in end-user groups. With respect to independent living, the aim was to demonstrate that clients can independently live longer and safer in their own home environment through the Assistive Technologies of ISISEMD. Furthermore, as ISISEMD develops assistive technologies and services, user satisfaction and acceptance were examined to assess how the end-users perceive the set of technologies as well as the services. More information for the evaluation framework for impact assessment of the services is provided in [21]. 

We defined the following research hypotheses:

(H_1_) if the personalised services offered by the ISISEMD platform are based on each client's specific needs, then the feeling of safety, ability for independent living in their home environment, hobbies, and lifestyle will have a positive impact on QOL;
(H_2_) if the services supporting the informal carers will reduce care-related stress then a positive impact on their QOL, in particular increased feeling of safety, and reduced rates of stress levels, will occur;
(H_3_) if the regional care providers will be able to offer social services to these groups of clients which are currently not included in the traditional care model, then there will be an increase in the access to and quality of social care.

Target variables and expected results: 

(a) increased QOL and feelings of safety, reduced care-related stress, and maintained cognitive ability are assessed by standardised questionnaires; 

(b) user acceptance and satisfaction are assessed by specifically designed ISISEMD questionnaires.

### 4.2. Validation of the Services

In the four pilot sites, the validation of the services was carried out in two stages—small-scale and large-scale validation. The services were first tested in a smaller scale, with a few end-users at each site for a period for 4 months, in order to identify if any technical problems existed before large-scale testing with all users during the rest of the testing period. This was also in ethical consideration as installing technology that may require adjustments could become a source of stress in the home of persons with CI. Additionally, to have multiple persons or multiple visits from project staff could also affect QOL, either positively or negatively. After that, the pilot operation and the controlled study continued in full scale, involving the rest of the test participants. The cities involved in ISISEMD study were Frederikshavn from Denmark (denoted as FRED), Lappeenranta from Finland (denoted as LAP), Trikala from Greece (denoted as TR), and Belfast from UK (denoted as BLF). Initially the overall period of real-life evaluation was planned for 12 months but it was later extended to 15 months.

### 4.3. Inclusion Criteria

The eligible population of elderly is: *elderly over 60 years of age diagnosed with stage two (Age Associated Memory Impairment) to four (Mild Dementia), according to the GDS, with corresponding to the Mini Mental State Exam (MMSE) scores of 19–26 and living in their own homes.*

To work with a representative sample of the primary end-users, the recruitment of ISISEMD trial participants for the pilot services follows strongly defined inclusion and exclusion criteria. The World Health Organisation (2007) International Classification of Diseases (ICD-10) was to be used to classify dementia type and used in conjunction with the Mini Mental State Examination (MMSE) [22] to determine cognitive status. The main inclusion criterion for primary users is the stage of disease (level of cognitive decline). The Global Deterioration Scale (GDS) was to be used as a classification standard. The main inclusion/exclusion criteria have been consulted with Bodil Gramkow, chief physician at Department of Psychological and Gerontology in Brønderslev, Denmark and Kasper Jørgensen from National Knowledge Center for Dementia in Denmark.

For assessing EP status, another mode was to administer the Montreal Cognitive Assessment (MoCA) [23] as an academic comparison tool for cognitive functioning measurements (sensitivity 90%) because MoCA is designed to screen for MCI and considers attention, concentration, executive functions, memory, language, visuo-constructional skills, conceptual thinking, calculations, and orientation in around 10 minutes. In this case, the MoCA score for inclusion would be less than or equal to 26 following the same cognitive functioning parameters as for the MMSE. 

*The eligible population of informal caregivers was adults over 18 years and they were recruited based on their relationship with an elderly test participant.*

Tables 1 and 2 summarise the inclusion and exclusion criteria for EPs and ICGs.

As our research progressed, we needed to make modifications to the inclusion and exclusion criteria. They are explained in Section 5. 

### 4.4. Statistical Considerations and Forming of Test and Control Groups

ISISEMD-controlled study on intelligent systems for dementia home care is a randomized control trial. Allocation concealment was implemented in order to assign the patients in the intervention and in the control arm of the study. The *intervention/test group* was provided the ISISEMD telecare platform while the *control group* received standard care services through their ICGs and municipality. Because of the nature of the intervention, masking of the intervention could not be performed (since the telecare platform was visible and some of its functions were interactive).The trial followed a single-blinded pattern in which the principal investigators conducting the assessments were not the researchers analyzing the data from the evaluations.

The overall hypothesis for ISISEMD project is that ICT services will improve QOL for those with cognitive impairments or mild dementia. The end-user partners are not aware of previous research to document such a hypothesis with a controlled study. Previous studies in this area have only found that there is a relation (Logsdon [24]). So, in reference with Logsdon [24], our assumptions are: 

*“…it could be expected an average score of QOL-AD at approx. 39.5 points (spreading 5.3) among a test group. In ISISEMD project, we will try the ICT service for N* = 97*.”*

We were aiming to be able to measure increased QOL with 6%, spreading 5.3 and *P* value of 5% is calculated that *N* = 37 persons to be used in the test group, in order to prove the hypothesis with significance. Because of this reason, the number of test elderly persons was defined to be *N* = 40 in the test group, meaning 10 persons in the test group and additionally 10 persons in the control group for each region. All in all, overall for the controlled study *N* = 80. Additionally, in order to have statically valid test results, the test and control groups of the elderly were planned to be randomly selected. This was to be done by a small lottery.

**Wilcoxon Test**

Please note that instead of regressions analysis, which is not a suitable test in our case due to the small number of test subjects, a Wilcoxon test was conducted. For small number of observations, we assume that distributions in scores and data are non-normal. The Wilcoxon Signed-Rank test detects differences in the distributions of two related variables. Small significance values (<.05) indicate that the two variables differ in distribution.

### 4.5. Ethical Aspects

When testing ICT services with human participants, it is necessary to obtain approval from national and regional Ethical Committees before the trials begin. This ensures that the ethical rights of the citizens are respected and that the testing is carried out according to the national and international regulations. For the ISISEMD controlled study, all required approvals from data protection agencies and ethical regional committees were obtained. Consent forms were signed by all persons in the study and details of the main person to contact from the social care provider organization were provided to all study participants. A short brochure with more information about the ISISEMD services was given, together with short description of the project and statement that all data is treated anonymously. As the ISISEMD pilot involved human participants, a number of ethical and legal considerations were considered and followed:

- *the right to be informed*: any participant in ISISEMD has the right to know the purpose of the activity they are involved in, the expected duration, procedures, use of information collected, their rights as a part of the study and any risks, discomfort, or adverse effects. This information was conveyed during the recruitment process and then reiterated when the *informed consent* form was distributed and signed by the participant;
- *permission to record*: before recording the voice or image of any individual, permission will be obtained through the consent form;
- *anonymity*: participants have a right to anonymity, meaning that their information was kept confidential and names were never associated with data or other personally identifiable information;
- *the right to withdraw*: participants should feel free to withdraw from any activity without penalty;
- *valid and reliable data*: in every activity, we ensured that the data we collected was free from bias, accurate, valid, and reliable;
- *data retention and documentation*: collected original data will be retained only for as long as it is relevant for the project.

In Greece, the Institutional Review Board (IRB) of the ISISEMD study was the Independent Authority of Personal Data Protection. In Denmark, the ISISEMD project was approved by the Data Protection Authority while the Local Scientific Ethics Committee for North Jutland Region was notified about the study with a full description of it and followed the rules set by the National Scientific Committee (and the Helsinki declaration). In Belfast, approval was sought from the Research Governance Department of the Belfast Health and Social Care Trust and the Northern Ireland Regional Ethics Committee and Lappeenranta obtained authorization from the local and national Social and Health Services and Ethics Committee. The IRB and the Data and Safety Monitoring Board were the same body.

### 4.6. Data Collection Tools

#### 4.6.1. User Acceptance and Satisfaction

In [25], Dillon and Morris present user acceptance as “the demonstratable willingness within a user group to employ information technology for the tasks it is designed to support.” Davis [26] developed the Technology Acceptance Model (TAM), which proposes that the acceptance of AT will be correlated with perceived value and ease of use. User acceptance and satisfaction, discussed in this paper from the perspective of the ICG, is assessed from both the technical and non-technical perspectives. By technical perspective, we mean that perspective in which the ICG finds the technology easy to use, accurate, and functional for their caregiving responsibilities. 

In ISISEMD, user satisfaction is evaluated by the end user's assessment of the multiple aspects of the service. This means that user satisfaction is considered a multidimensional concept, incorporating the perceptions of end users based on their personal, subjective attitudes and values. To collect data, we used a triangulation of methods consisting of questionnaires, interviews, and structured observations with the users to determine their acceptance and satisfaction with the ISISEMD services. ISISEMD questionnaires for user acceptance and satisfaction have been inspired by The Quebec User Evaluation of Satisfaction with Assistive Technology (QUEST 2.0) [27, 28] and ETUQ—Everyday Technology Use Questionnaire [29]—and were administered to both EP and ICG groups. 

#### 4.6.2. Quality of Life for Informal Caregivers

There are few instruments measuring QOL for caregivers of chronically ill patients. A more recent and advanced assessing tool for measuring QOL of informal caregivers, specifically designed for carers of patients with cognitive problems, is the Scale of Quality of Life of Caregivers (SQLC) [30]. This tool was used in the controlled study, covering 3 domains: professional activities, social and leisure activities and responsibilities of caregivers to help patients in everyday living. SQLC scoring provides 4 categories of Caregivers' adaptation: full psychosocial adaptation (141–145), mild disturbance (100–140), moderate disturbance (86–99) and severe disturbance (<85). 

#### 4.6.3. Stress of Caregiving

Assessing caregiving stress involves an evaluation of how the caregiver experiences the caregiving task to be, involving objective parameters (e.g., number of tasks, time per task) and caregiving capacity (e.g., amount of available time, proximity to care receiving residence), among others. To measure caregiver stress as well as effects from interventions aimed at reducing it, the Zarit Burden Interview (ZBI) short version [31] was used in ISISEMD. Questions are on caregiver's health, psychological wellbeing, finances, social life, relationship between carer and patient, and a lower score indicates lower perceived stress.

More information about the overall evaluation framework for ISISEMD services with description of the mentioned data collection tools is presented in [21].

### 4.7. Data Collection Methods

Collection of the study data was carried out at three stages—at baseline, at intermediate, and at final stages. The pilot started in May, 2010, and final data collection took place in June, 2011. It must be noted, though, that the pilot services have been used with different duration in the regions—in Lappeenranta and Frederikshavn since May, 2010, in Belfast since July, 2010, and in Trikala since September, 2010. The intermediate evaluations that we describe in this paper took place in February, 2011, while the baseline evaluation for the clients was carried out before the services were installed in their homes. The participants that carried out the intermediate evaluations in February, 2011 were those who were using the services long enough in order to get used to the technology and to observe a difference in their every day. Their number is provided in the sections for the primary and secondary outcomes of this study.

The intervention and the control groups of EPs and ICG were administered the same ratings scales and questionnaires except the user satisfaction and acceptance. The rating scales for SQLC and ZBI for ICG were administered at baseline, intermediate and final stage for the intervention group, while for the control group—the same rating scales but only at baseline and final stage. In this paper we present the baseline and intermediate results from these for the test group. For EPs, the rating instruments used were MMSE, ADL, IADL, and QOL-AD but the results from them are not the subject of this paper. 

## 5. Results

### 5.1. Recruitment of Participants

The test participants from the four countries were recruited using different channels. The majority of referrals were contacted via memory clinics or by the home-care personnel. But we also used TV and radio channels to announce the pilot study and attract interested participants. 

The control EP group was as characteristically similar to EP group as possible. Participants were also recruited with the aid of general and nurse practitioners, memory and dementia clinics, and regional organizations working with dementia populations. They were going to be involved as the test participants from the beginning months of the test period and relevant tests were applied to them too. Control group was also administered all tests for the test group except test user acceptance and user satisfaction because they were not given any technological intervention.

As the regional end-user organizations began to identify potential clients, we have had to make amendments in our inclusion criteria and parameters. As none of the care provider organizations were familiar with the GDS scale, it was determined that we would classify the level of cognition based on MMSE scores only. Likewise, not all participants had a medical diagnosis of dementia and accompanying stage, many were referred by professionals who noted cognitive impairment and probable dementia or by their relatives. Since we were not using the GDS as our measurement, we no longer were restricted to the 19–26 score range in the cognition scales. We observed that the elderly participants from the target group, in most of its majority, were not receiving any dementia care because this type of care is usually offered to clients with severe dementia. They were mainly known to the FCGs from receiving traditional home help. We also noticed that overall, there is little awareness in the society about the early signs of dementia and the care stress of the informal caregivers and that the early stages of illness are not diagnosed. A fact that is also confirmed by a recent study in UK from the Alzheimer's Association. 

Additionally, we had initially specified that we would exclude participants who have dementia secondary to head trauma as well as those who are bedbound (confined to a bed or chair for 20 hours a day for 4 out of 7 days). In Region Frederikshavn, there was one EP who has cognitive impairment secondary to head trauma and is confined to a wheelchair due to a work (fishing industry) accident. The case of this participant was discussed with the other care provider organizations and it was determined that this subject and the informal caregiver would remain in the trial because they are only utilizing the Lommy (GPS) service and both EP and ICG give valuable feedback regarding the ISISEMD equipment and services. It is also noted that the main constructive outcomes in this case will be in the evaluation of services and QOL rather than in the correlation between cognitive functioning. There was also one participant that was not willing to take the tests for the cognitive impairments, neither at the baseline, nor at the final evaluations because he felt offended by the questions. Furthermore, there were some EP subjects with MMSE scores outside range 19–26. From the intervention group, there were *N* = 3 with MMSE   <   19, *N* = 2  with MMSE >  26. From the controls, *N* = 5 with MMSE < 19, *N* = 7 with MMSE   > 26.

### 5.2. Number of Participants

The goal of the controlled study was to include 80 elderly patients with MCI or mild dementia (MD) across four regions—20 per trial site (10 intervention and 10 control participants) with respective number of informal caregivers. 

However, it must be noted that it was a very challenging task to recruit test participants for the study due to several reasons. The partners from the regional organizations invested a lot of effort in these activities and normally two to three times more referrals have been approached and interviewed in order to fit the inclusion criteria. Also, due to some procedural delays in some of the regions for obtaining ethical approvals and appointing staff to work on the project, the pilot operations did not all start at the same time in all of the four pilot sites. There were also issues with some participants experiencing a rapid change in cognitive or health status that excluded them from the trial by the time they began, which is not uncommon in this field of research. Therefore, in the selection procedure we could not follow the randomization of the test and control participants. Priority was given to include the target number of participants in the intervention group, therefore the controls were recruited after the recruitment of the intervention group. In Frederikshavn pilot site it was not possible to recruit any participants to be in the control group. 

Table 3 presents the number of subjects from the intervention and control group and the number of drop outs from the intervention group. The most common reasons for drop outs were that the overall health status degraded significantly and elderly were admitted to institutional care, or participant had a stroke and was not able to continue the pilot or the services could not be installed in their homes due to problems to provide internet connection. Another reason was that after signing the consent agreement forms the baseline tests were administered but there was a sudden change in family plans and the drop-out took place before the equipment was installed. Other reason for dropouts included family issues. Only one dyad dropped out from the intervention group due to the number of false alarms during the initial months of the small-scale pilot and because EP considered their own health good enough to manage without trying the services.

### 5.3. *Participant* Characteristics

#### 5.3.1. Characteristics of the ICG Sample

In total ICGs *N* = 71 participated overall in the control study, with *N* = 45 in the intervention group and *N* = 26 controls. From the intervention group, there were *N* = 14 dropouts due to a drop out of the elderly. Overall, the female ICGs were 70.89%, 64.86% intervention, and 76.92% controls. 67.56% of the intervention group had a previous experience with computer, while 83.78% of the intervention group had a previous experience with a mobile. 82.28% from all of them lived in the same area as their elderly with 83.78% from the intervention and 80.77% from the control. The percentage of the children was highest—57.80% overall (54.05% intervention and 61.54% from the control). The second high was the percentage of the spouses—41.22% overall (32.43% intervention and 50% from the control), the rest were other type such as neighbors or some volunteers who were helping the elderly. Mean age, in years in the intervention group was 54.89 years (*N* = 27, SD 12,939) while the control group had 62.23 mean age (*N* = 26, SD 13,131). 

#### 5.3.2. Characteristics of the EP Sample

In total EPs *N* = 71 participated overall in the control study, with *N* = 45 in the intervention group and *N* = 26 controls. From the intervention group, there were *N* = 14 dropouts due to reasons mentioned above. Overall, the female EPs were 64.55%, 67.6%, intervention and 61.5% controls. Only 8.1% of the intervention group had a previous experience with computer, while 51.35% of the intervention group had a previous experience with a mobile. 47.6% from all of them lived alone, with 56.7% from the intervention and 38.5% from the control. Mean age for EPs, in years, in the intervention group was 77.38 (*N* = 37, SD 8.060) while the control group had 80.00 mean age (*N* = 26, SD 8.23). 

The cognitive functioning for EPs, depicted with MMSE scores, was: for intervention group mean = 22.12 (*N* = 34, SD 3.79) and for the control group mean = 22.29 (*N* = 24, SD 5.44).

After analyzing the data from the regions, we found that that in the intervention group (*N* = 37), the most EPs living alone were in LAP (90%, *N* = 10) and in FRED (70%, *N* = 10) and the least were in Belfast (14%, *N* = 7), in TR they were 40% (*N* = 10). 

Overall in the intervention group (*N* = 37), the male EPs were less than the female EPs with highest percentage in FRED (50%, *N* = 10) and the smallest percentage in LAP (10%, *N* = 10), while in BLF it was 43% (*N* = 7) and in TR it was 30% (*N* = 10). 

### 5.4. Use of the Services

**Figure 2:**
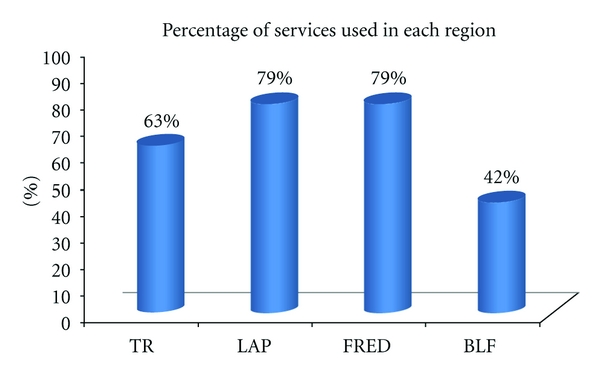
Average use of the services in the four cities (*N* = 31).

From all services that were offered via ISISEMD service platform and available during the test period of the controlled study, not all of the services were tested in each home because each elderly received a subset of all services deepening on his and relative's individual care needs.  Figure 2 shows the average use of the services in the four sities. To identify which service each elderly needed at baseline, an evaluation of basic activities of daily living (ADL) and the instrumental activities of daily living (IADL) was carried out, together with an interview with the informal caregivers about their needs and if the elderly had any incidents recently (such as being lost, any falls, cooker turned on and forgotten). 

### 5.5. Observations from the Baseline Evaluations

We would like to summarize the following observations from the baseline evaluations:

- highest percentage of services utilized was in Lappeenranta (Finland) and Frederikshavn (Denmark);
- trikala (Greece) had the most end users living alone (100%);
- most of the EPs and ICGs were women, which follows global trends for these groups;
- according to MMSE scores, in Belfast (UK), EPs have moderate impairment, while the rest is in mild to moderate stage;
- in Lappeenranta (Finland) and Frederikshavn (Denmark), ICGs report a “moderate effect” on QOL before the intervention, while in Trikala, Greece and Belfast, North Ireland, the effect on ICG QOL was reported as “severe”;
- the highest reported caregiving-related stress for ICG is in Frederikshavn (Denmark).

In the following sections we present more detailed information about the results from the intermediate evaluations. 

### 5.6. Primary Outcomes: Quality of Life and Care-Related Stress for Informal Caregivers

The participants that carried out the intermediate evaluations in February, 2011 were only from the intervention group and those who were using the services long enough in order to get used to the technology and to observe a difference in their every day. Their number is provided in each of the tables for the primary and secondary outcomes of this study.

Table 4 provides data about the nature of the caregiving relationship to the older adult, QOL in the ICGs via SQLC and reported level of care-related stress through the ZBI scores from baseline and intermediate assessments.

SQLC and ZBI rating scales were administered among ICGs at the intermediate evaluation in all three pilot sites except Frederikshavn.

Table 4 is showing that, on average, the adaptation of the caregivers is within the same range—but that range is also the lowest, showing severe disturbance to ICGs QOL. However, we can observe that the median (middle score of all assessments) SQLC score increased, from severe disturbance to one of moderate disturbance. As well, the mode (most commonly reported assessment result) increased from severe to moderate as well as the maximum score increased for the follow up. 

We can also observe that the average (mean) level of caregiver stress dramatically decreased from 31.82 to 14.83, meaning from on the moderate side to little to no caregiving-related stress. The median (middle score) also decreased as well as the most reported score (mode) and the maximum level of stress reported dramatically decreased (from 105 to only 24 being the highest reported level of stress).

SQLC score at baseline (*N* = 27) had mean value 81.96 (SD 21.283), with minimum = 29 and maximum = 113. SQLC score at intermediate (*N* = 13) had mean value 73.46 (SD 30.341), with minimum = 26 and maximum = 120. This shows, again, that the reported QOL of ICG has decreased.

**Figure 3:**
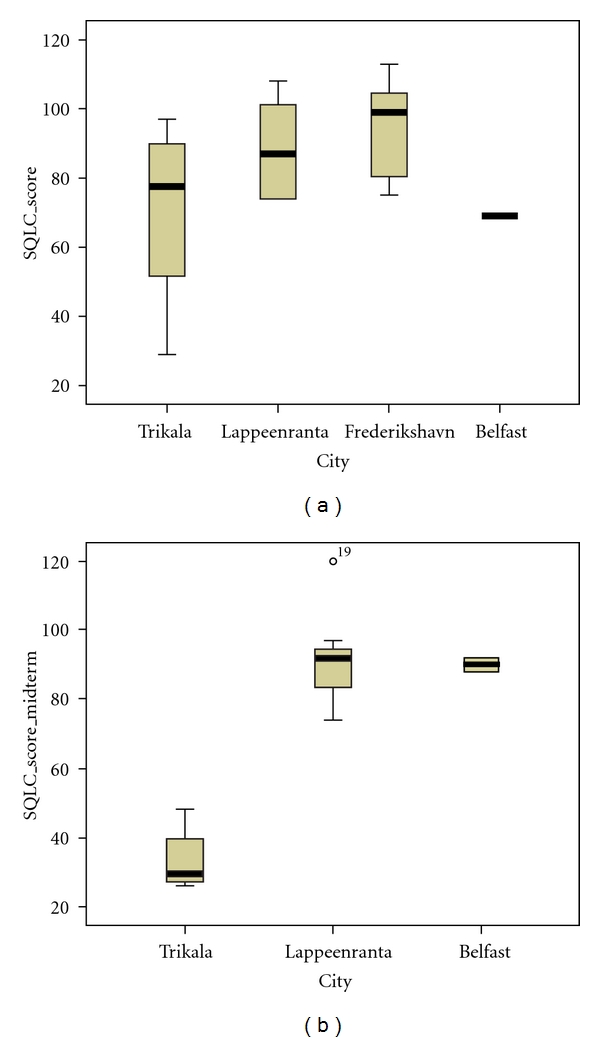
Boxplots of SQLC scores (baseline *N* = 27, intermediate *N* = 13) according to city.

Boxplots of SQLC scores according to city are presented on Figure 3. Frederikshavn had the highest baseline SQLC score, while Trikala and Belfast had the lowest baseline SQLC score. In intermediate scores, Trikala seems to be by far the lowest scoring city in SQLC, while Lappeenranta and Belfast seem to be close in scoring; there are no available intermediate SQLC scores for Frederikshavn. In terms of difference between baseline and intermediate SQLC scoring, Trikala seems to be the city with the largest drop in scores, while Lappeenranta's scoring seems to range in the same levels; Belfast seems to have small increase in SQLC scores, however there are only few data available for intermediate scoring in this city.

**Figure 4:**
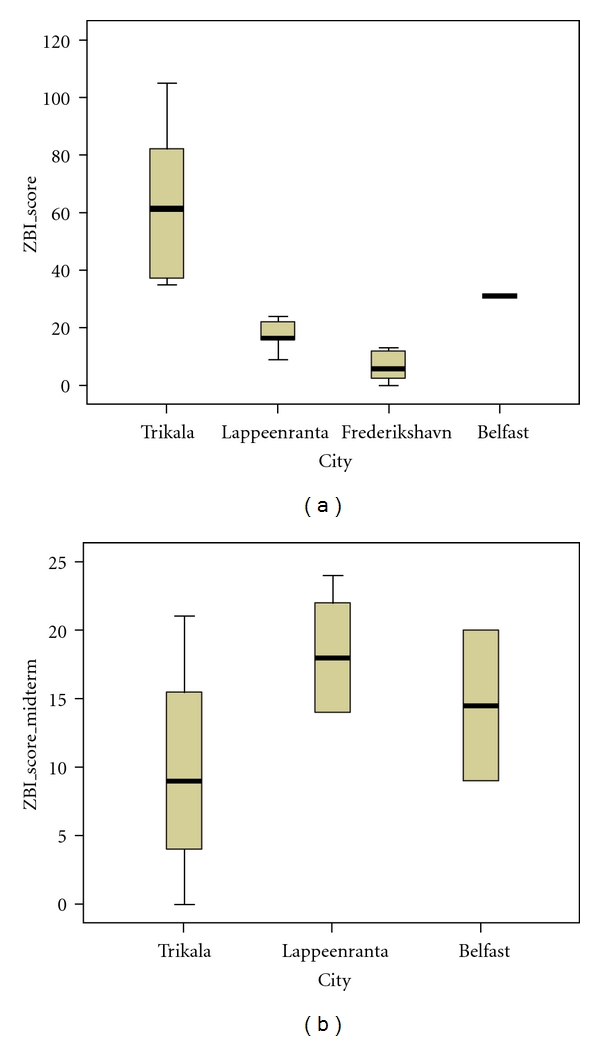
Boxplots of ZBI scores (baseline *N* = 28, intermediate *N* = 12) according to city.

Boxplots of ZBI scores according to city are presented on Figure 4. Trikala seems to have the highest baseline ZBI scores, while Lappeenranta and Frederikshavn seem to have the lowest baseline ZBI scores. At intermediate scores, Trikala seems to be the lowest scoring city in ZBI, while Lappeenranta and Belfast seem to be relatively close in scoring; there are no available intermediate ZBI scores for Frederikshavn. In terms of difference between baseline and intermediate ZBI scoring, Lappeenranta seems to be the city with the largest increase in scores, while Trikala's scoring seems to range in the lowest levels; Belfast seems to have a decrease in ZBI scores.

ZBI score at baseline (*N* = 28) had mean value 16.06 (SD 8.11), with minimum = 0 and maximum = 33. 

ZBI score at intermediate (*N* = 12) had mean value 14.83 (SD 7.095), with minimum = 0 and maximum = 24. This shows, again, that the reported care stress of ICG has decreased. 

Table 5 examines the normality in the distribution of values for the SQLC and ZBI scales (baseline, intermediate). When *P *value <0.05 the values do not follow a normal distribution (this determines the choice of the statistical analysis that we are using: when variables are not normally distributed we use nonparametric methods). In our case, if we use the first statistical criterion (Kolmogorov-Smirnov), the distribution is nonnormal and due to our small sample (small number of observations), we assume that distributions in scores and data are nonnormal. For the above reason we run a Wilcoxon test, as it was not possible to conduct a regression analysis (the criteria for using regression analysis are not met).

Table 6 provides results from the Wilcoxon test for SQLC, ZBI. The Wilcoxon test examines whether the SQLC scores show significant differences at intermediate evaluation: The significance, “Sig” (*P* value), which is larger than 0.05 (*P* = 0.153) shows that the differences are not significant (even though at baseline the Mean was 81.96 and at intermediate it was 73.46).

Similarly, Wilcoxon tests for ZBI and SQLC scores found no significant differences between baseline and intermediate (with *P* = 0.123 and *P* = 0.536, resp.).

For these two (SQLC and ZBI), showing *no significant difference*, we would like to state that this has, statistically, shown that ISISEMD services can at least maintain QOL in family caregivers.

It appears that the lack of sufficient data at present does not permit us to conclude that there is a significant difference (in either direction) between baseline and intermediate.

### 5.7. Primary Outcomes—User Satisfaction

Intermediate evaluation for user satisfaction with the services was carried out with 17 EPs and 17 ICGs from the intervention group from the four regions and gave positive results overall—on average 70.45% for EP and 65.12% for ICGs and 88.25% of both groups indicate that they would like to continue to use the home support services after the end of the project. Access to care for elderly was increased with 100% for all test elderly persons because they did not receive this type of care before. Another positive effect of using the services for the informal caregivers is that they give them possibility to save time and money on travel and phone calls to the elderly, gives them more freedom for their personal life and free-time interests, reassurance and peace of mind.

**Figure 5:**
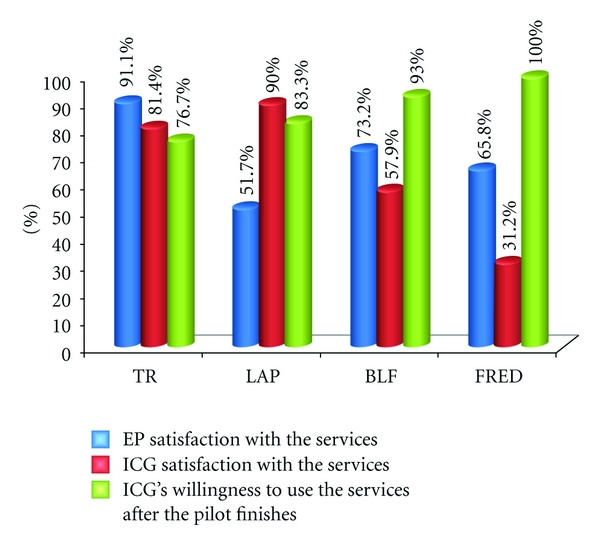
User satisfaction results from the intermediate evaluation—overall for EP and ICG subjects (intervention group).

Figure 5 presents the results from user satisfaction and willingness to use the services after the pilot finishes, in percentage for the four regions separately. For satisfaction 100% = full satisfaction, 0% = not satisfied; for willingness to use 100%= I would definitely use a system like this, 0%= I would not use a system like this. They are based on the flowing number of subjects: TR-EP *N* = 4; ICG *N* = 4; LAP-EP *N* = 7; ICG *N* = 7; BLF-EP *N* = 2; ICG *N* = 2; FRED-EP *N* = 4; ICG *N* = 4.

It must be noted that, from a technical perspective we faced some unexpected technical issues with stability and availability of the services in real-life conditions during the first couple of months in Frederikshavn and Belfast which influence the results from this intermediate evaluation and thus shows lower percentage of satisfaction. For the final evaluations, we will be investigating correlations between particular age group of ICGs and their changes (using regression analysis) and correlations between living status of ICGs to EPs.

### 5.8. Secondary Outcomes—The Services Are Appreciated Differently in the Four Regions

During the intermediate stages of the project, when we were designing the needed services, the care provider organizations and end-users from all four pilot sites participated in collecting user requirements. So the list of services to be technically implemented in ISISEMD system was a collective list, addressing the overall needs. However, due to country specifics, as we found out already from the intermediate qualitative evaluations, some services were more popular (the end-users liked them most and /or considered them most important for the care provision and quality of life) in some of the regions than in the others. Here we presented the specifics we observed:

- EPs from Frederikshavn, Belfast, and Trikala are more interested in having the services for outdoor positioning from *Lommy*, compared to users in Lappeenranta;
- from home safety services—*fire alarm* and *cooking monitor* are considered most valuable for care-giving and personal safety in Frederikshavn, while in Lappeenranta, these are the *Intelligent front door* and *Person out of home for too long service*;
- elderly users from Frederikshavn, Belfast, and Lappeenranta appreciate *Memory Lane* service because it gives them something very personal and facilitates conversations with family and other visitors;
- *date and time services* are appreciated by EP in all regions as they reinforce the structure of the day and gives the elder the feeling that they manage better in daily routines, giving them more independence;
- elderly and relatives find the *Reminders service* helpful with reminder prompts, and even more the prompts by an external person reduced the stress and aggressiveness in the relationship EP-closest family member since EP was more prone to listen to the reminders coming from the system than from the family member.

### 5.9. Secondary Outcomes—Positive Influence for the Informal Caregivers and the Elderly Persons

Despite of the quantitative results from the intermediate evaluations, positive socio-economic effects were experienced by both EP and ICG. 

There are indications for the following positive influence for the informal caregivers:

- reduced number of phone calls by ICG to check on EPs condition;
- reduced number of visits done by ICG to check on EP's condition;
- reduced number of times ICG discusses every day issues related to the illness with EP;
- increased possibility for ICG to do free time or personal activities out of house;
- reduced time spent to drive/travel to visit the EP.

There are indications for the following positive influence for the elderly:

- EP goes out of house on him/herself more often (reduce social isolation);
- reduced number of times per day EP calling on the phone to ICG;
- reduced number of times per day EP asked ICG for future events.
- reduced number of times per day EP asked ICG for day/time orientation.

Additionally, in ISISEMD controlled study, we have found that the end users themselves provide much more information as to dementia care, caregiving and independent living than we had anticipated. Some end users find new uses for the technology or equipment that was not envisioned by the developers as well as presented useful feedback on the functionality of the system. Examples of this are further described in the appendix with the qualitative results from the study.

## 6. Discussions of the Results from the Intermediate Evaluations

The ISISEMD European project aims to improve the care and Quality of Life of elderly persons with cognitive problems or mild dementia, while evaluating the care services provided. In the context of this project, during the intermediate evaluations, 17 informal caregivers (ICGs) from 4 different geographical regions (Greece, Finland, Denmark, and UK) and their relatives with CI have been asked to evaluate the care services provided, in relation to the new assistive technologies, as well as the impact these have on their everyday life. The Scale of Quality of Life of Caregivers (SQLC) and Zarit Burden Interview (ZBI) evaluation were employed at baseline and at intermediate stages. The intermediate evaluation was carried out after an initial test period of 6 to 10 months. 

During the whole process of piloting the services in real life, we observed that even when EPs and ICGs were skeptical in the beginning, after giving them time to get used to the technology, the elderly and their relatives accept the technology and can see the opportunities for positive impact. Since the targeted population have cognitive impairments or mild dementia, we found that it is most beneficial if the services are introduced as early in the disease progression as possible and it takes about 4–6 weeks for EP to get used to the system. Of course, during this period, some effort is needed from ICG to remind them to refer to it. It also helps if the Carebox touch screen is placed close to a TV (a device to which all elderly are used to) or such place in the house where the elderly spends most of the time. The deferent levels of interaction with the system, presented in Section 3, also help for the individual acceptance. The Memory Lane service was very much appreciated by the elderly and it was also a point of reference to the Carebox. Since the service platform is quite flexible, a number of settings can be made in order to adjust the services to the progression of the dementia. For example in the initial period of having the system, the Help button on the Carebox can be visible and EP can get in contact with ICG. Later, it can be “removed” from the Carebox. All in all, if EP cannot interact with the system, this is not needed but the home safety services keep ICG informed about the safety of EP.

In most of the cases, the family caregivers prefer receiving notifications and alarms by SMS instead of by email. Family caregivers are also less sensitive to the service cost compared to elderly. It seems that they are interested in having such a solution in the home in order to increase the level of independent living of their relatives. This is mainly due to the fact that relatives are in the middle age and they have a higher income in comparison with the elderly, and also due to the ICGs having spent more time and money on technology than the elderly EPs. In addition, relatives would like to have a system that offers a high degree of independence for the elderly. 

The automation degree of the services is also of a key importance for the family caregivers, as we found out. Informal caregivers were also willing to have very few degree of interaction with the system. This is mainly due to the fact that they understand, on the one hand, that elderly is not able to be familiar with the new technology and on the other hand, they are willing to increase the level of independence of the elderly. Their busy everyday life also plays a role. In addition, elderly do not want a lot of user interactions with the system, since they are not familiar with the new technology and have somewhat of an aversion to learning. We have also seen that this may discourage use of the system, as the EPs may worry that they will break the technology, so they would rather not use it at all than be the cause of expensive repairs. The EPs want the system to be as automatic as possible; however, they want inexpensive technological solutions. 

According to the statistical analysis of the available data, no significant differences are shown in the evaluation scores provided by ICGs at the intermediate evaluation after 6–10 months from the baseline period. However, we can say that this has, statistically, shown that ISISEMD services can at least maintain QOL in family caregivers and alleviate care-related stress. A relatively high satisfaction with the services is shown by both the elderly and the informal caregivers. Moreover, the qualitative feedback from the elderly and the informal caregivers depicts positive impacts from using the ISISEMD services. 

New data are expected to become available from the final evaluation in June 2011, also including the rest of the test participants. We hope for that the final evaluation, involving around 60 EPs and 60 ICGs from both intervention and control group would allow us to support our hypothesis with a statistical significance. 

## 7. Conclusions

As one of the formal caregivers from ISISEMD consortium stated, *“it is quite a challenging task to design technology services to support persons with dementia living at home and their caregivers because it is not like designing services for physically handicapped persons.”* Part of the challenges comes from the fact that each person with dementia has specific individual needs depending on how the disease progresses and on the support he or she gets from the closest family. The use of technology as support for persons with dementia living at home sets different requirements for the development of services. Different kinds of technology solutions are needed depending on individual personal factors. Furthermore, it is important that the system works with a minimal interaction and with automated operations because of limited learning abilities among the users or because they have very little experience with the new technologies. 

We would like to thank our test users whom we accept as an equal partner of the consortium. They played a very important role in the process of bringing the services to a mature level and improving them in all aspects in order to meet their needs as best as possible. We can confirm that it is of high importance that the primary user and caregivers to be motivated towards usage of aiding technologies in their homes. For the acceptance of the services by the elderly, a key role plays their family caregiver and the process is much faster and easier if the caregivers have previous experience with technology.

We know that skeptical users are stoppers against introduction of new technologies. But our experience shows that the elderly and their relatives accept the technology and can see the opportunities for positive impact and added value from the use of the services in their everyday life after giving them time to get used to the technology, even when EPs and ICGs were skeptical in the beginning. It can be expected that after about one month, the elderly and the family caregiver can get used to the services. The most successful adoption of the services can happened when they are offered as early as possible in the history of the disease—in this way the technology services can be integrated in the coping and care strategies in the family and the elderly has highest chances to learn to refer to the Carebox with the reminders and to use the Lommy device. 

## Figures and Tables

**Table 1:** Inclusion and exclusion criteria for EPs.

Factor	Age	Medical diagnosis	Living arrangement	Caregiver
Inclusion	Over 60 years old	GDS Stage 2–4; MMSE and MoCA score of 19–26	Lives in home dwelling	Have ICG

Exclusion				
		GDS beyond Stage 4; dementia secondary to head trauma; bedbound; malignant illness; psychological conditions similar to dementia; misuse of alcohol or medications; frontal temporal dementia; more than 3 acute hospitalizations in the past 12 months	Planned long-term care admission in 6 months or less	Do not have someone in the role as ICG
				

**Table 2:** Inclusion and exclusion criteria for ICGs.

Factor	Age	Medical diagnosis	Living arrangement	Caregiving
Inclusion	18 years or older		Live with EP;	Actively involved
			Do not live with EP but provides regular, direct assistance; No plans to move for the duration of the trials	In caregiver role for 6 months or longer

Exclusion		Active treatment for cancer; has dementia	Planned placement of EP in long-term care in 6 months or less	

**Table 3:** Number of subjects participated in ISISEMD-controlled study.

Region	EP intervention (*N* = 31)	EP (intervention) dropouts since start (*N* = 14)	EP controls (*N* = 26)	Total EP subjects until end of pilot (*N* = 71)
FRED (Denmark)	6	4	0	10
BLF (UK)	7	5	10	22
TR (Greece)	10	3	5	18
Lapp (Finland)	8	2	11	21

Region	ICG intervention (*N* = 31)	ICG (intervention) dropouts since start (*N* = 14)	ICG controls (*N* = 26)	Total ICG subjects until end of pilot (*N* = 71)

FRED (Denmark)	6	4	0	10
BLF (UK)	7	5	10	22
TR (Greece)	10	3	5	18
Lapp (Finland)	8	2	11	21

**Table 4:** ICG percentages, SQLC and ZBI baseline and intermediate scores.

	ICG: Child	ICG: spouse	ICG: other	SQLC baseline	SQLC intermediate	ZBI baseline	ZBI intermediate
*N* observations	35	35	35	27	13	28	12
Missing	0	0	0	8	22	7	23
Mean/Percentage	62.9%	20.0%	17.1%	81.96	73.46	16.06	14.83
Median	—	—	—	85.00	88.00	21.00	15.00
Mode	—	—	—	74	92	16	14

**Table 5:** Tests of normality.

	Kolmogorov-Smirnov(a)			Shapiro-Wilk		
	Statistic	df	*P*-value	Statistic	df	*P*-value
SQLC_score	0.195	8	0.200	0.869	8	0.147
SQLC_score_intermediate	0.209	8	0.200	0.838	8	0.072
ZBI_score	0.275	8	0.077	0.831	8	0.061
ZBI_score_intermediate	0.174	8	0.200	0.951	8	0.721

**Table 6:** Wilcoxon test for baseline and intermediate results of the Scale of Quality of Life of Caregivers and Zarit Burden Interview.

	SQLC score intermediate—SQLC score	ZBI score intermediate—ZBI score
*Z*	−1.428	−1.542
Asymp. Sig. (2-tailed)	.153	.123

## Appendix

### Qualitative Results from Service Use

During the pilot operation, the researches collected a number of good stories from the test participants, showing how the services helped the elderly (EP) or informal caregivers (ICG) in their daily life or how the users adopted the services and used them in their own way to fit individual needs and desires. Some of the examples are listed below, showing the results from the qualitative evaluations.

(i) Help Button on the Carebox One of the difficulties EP faces is to use the telephone to call relatives. She is now used to using the Help button on the Carebox to make a contact with them. When she wants to get into contact with her ICG, she presses the Help button on the Carebox and makes very easy contact as the help button sends a text message to the family caregiver's mobile phone. If the ICG is at work, he is notified of this but in such a manner that it does not cause alarm. Although this service of the Help button was designed to be an easy way to contact someone if the elderly person needs immediate help, this particular user and her family have found that the easy functionality replaces her need to use the telephone, which has become too difficult for her.

(ii) Free Text Reminders on the Carebox One ICG uses the free text reminders to write to their EP to perform different small tasks. “It is like sending a SMS (text message) to the elderly but it is shown in larger letters on the Carebox because elderly cannot read SMS.” Another ICG uses the free text service to reinforce family humor, writing inside jokes or anecdote to EP such as, “have a glass of wine with your evening meal.”

(iii) ISISEMD Services Overall The formal caregivers observed that the services improve communication and relations among the elderly and family caregivers. The ICG can upload pictures on Memory Lane service to be seen by elderly, which also gives them something to discuss when they are together, “Did you see the pictures of your anniversary party on the Memory Lane?”. Moreover, the reminders “spoken” from the system help reduce the stress in the relationship among the elderly person and the family caregiver

(iv) ISISEMD Services Overall One family carer who is the son of a female test person noticed savings of his time and travel costs. In his mother's home, home safety services were installed (fire/smoke sensor and cooking monitor), together with the Carebox touch screen. Before installing the services, he was driving to visit his mother several times a week, but now he can see her activities via the ISISEMD web portal, and knowing that the home safety sensors will send him sms in case of smoke/fire or too long cooking activity, he feels much safer and does not need to drive there so often; the distance to her home is 7 kilometers. He does not worry all day long anymore because she knows how to contact him through the Help button on the Carebox if she needs anything. Only first after using the services, had the son realized how stressed he was in caring for his mother and the benefit that he gets from using the services is beyond his initial expectations. Furthermore, with dementia progression, he noticed from the overview of the daily events that his mother does not cook any more during the week days. For preventing of losing more weight, the son made everyday reminders to his mother via the Carebox for having regular meals.

(v) Outdoor Safety with Lommy Another elderly who is living alone is suffering from memory problems and severe diabetes for several years. He must walk every day in order to get the blood pressure in his feet down. Before he received the ISISEMD services, there has been an incident when he left home and felt very confused during his walk and a person in the neighborhood helped him find his way home. Last winter he was not walking outside at all because he was afraid to get lost, based on this previous experience. But this winter, even though there was quite a lot of snow in Denmark, he is walking the dogs of his neighbors several times per day because he has the Lommy and feels safe. These neighbors, as informal caregivers, can “see” his position on the map on ISISEMD web portal when he walks the dogs. He feels safe having the Lommy along on the trips. Due to the traumatic experience he had in becoming lost before, he always keeps the Lommy in his pocket. When he is in the home, the cord to charge the device is connected to the Lommy, which remains in the pocket at all times. In this way he never forgets it.

(vi) Cooking Monitor In the end of February, 2011, when the system was under optimization, a formal caregiver received a text message with “Cooking activity has been too long” (that is around 60 min of cooking activity)for one test person. It was afternoon and she called the test person and found out that the woman began to cook, but felt tired and lied down on the coach and fell asleep, forgetting the cooker. When the formal caregiver called the test person on the phone to check if everything was fine, she woke her up and the elderly switched the cooker off. Usually after 60 minutes of cooking, the Carebox would also “tell” the elderly about this alarm, but on this day, the audio feature was turned off due to the testing.

(vii) Out of Bed for Too Long One elderly had several incidents where she has fallen down during the night. Her relative had received the SMS message where it was written that EP has been out of bed too long. The ICG went to her mother's home first thing in the morning and found her mother fallen down on floor and could not get herself up. The daughter is very happy that she got the text messages, even though she got them during the night.

(viii) Person Left the House One EP is not safe to leave the house by herself anymore and the daughter, as ICG, received text messages from the ISISEMD system that her mother had left the house. The daughter went to her mother's place and found her walking on the street, confused and without knowing where to go. Particularly in Finland, this service is very important since during the winter it is very cold and dangerous for the elderly to go out of home for an extended period of time.

(ix) Intelligent Front Door Two of the test EPs has started to receive help from local homecare services. The relatives can see on intelligent front door service when the homecare nurse has visited and for how long. The relatives have been very satisfied with this service, because they can be sure that nurse has visited as it had been agreed upon.
